# How the glucocorticoid receptor contributes to platinum-based therapy resistance in solid cancer

**DOI:** 10.1038/s41467-021-24847-6

**Published:** 2021-08-16

**Authors:** Dorien Clarisse, Karolien De Bosscher

**Affiliations:** Translational Nuclear Receptor Research, UGent Department of Biomolecular Medicine, VIB Center for Medical Biotechnology, Ghent, Belgium

**Keywords:** Cancer therapeutic resistance, Chemotherapy

## Abstract

Synthetic glucocorticoids serve as co-medication against solid malignant tumors. However, glucocorticoid receptor activation may promote unsolicited cancer resistance to chemotherapy. The Kang team elucidated a glucocorticoid receptor-centred chemotherapy-resistance mechanism to cisplatin and characterized avenues towards a viable escape strategy.

Platinum-based compounds, such as cisplatin or paclitaxel, are in use since many decades for the treatment of solid cancers, including tumors of the lung, cervix, ovary, breast, head, and neck. In the active treatment phase, chemotherapy-induced nausea and vomiting are among the most dreaded adverse effects for cancer patients. In the past 40 years, synthetic glucocorticoids such as prednisone and dexamethasone were highly welcome co-medicated drugs to relieve all sorts of allergic reactions to the chemotherapeutic cocktails, and not in the least to overcome a debilitating hyperemesis^1^.

Over a decade ago, glucocorticoids turned from chemotherapeutic allies almost into enemies when evidence accumulated that the very same drugs that make chemo treatment tolerable to patients were counteracting the antitumor action of paclitaxel- or cisplatin-based chemotherapy^2^. This news came as a bombshell and was not an isolated event, as similar findings were reported soon after for glucocorticoids in the context of resistance to antiandrogens^3^. Glucocorticoids are used in the clinic since their discovery in the 1940s. Spectacular results in treating rheumatoid arthritis patients justified Nobel prize awards to Hench, Kendall, and Reichstein. At the time, their use was purely on an empirical basis, without in-depth knowledge of the underlying action mechanism. It was not until 1985 that one of the corresponding target proteins, the glucocorticoid receptor, was cloned. The glucocorticoid receptor has a widespread distribution in the body and combines many different functions, ranging from development to immune system regulation to metabolism. Despite the many advances on the diverse and cell-dependent action mechanisms of the glucocorticoid receptor by many researchers worldwide, the reality is that still today, we only partially understand how this intracellular receptor, annex transcription factor, selectively regulates its many different target genes. In the past three decades, the number of different molecular mechanisms that have been described to explain glucocorticoid receptor action almost seems to match the high number of cell and tissue types where this ubiquitous receptor can be found^4^.

Glucocorticoids are well known for their immune-modulatory actions. Their activity on the immune system occurs in a highly cell type-specific manner, reflected by variations in the extent and direction of the transcriptional response, indicative of mechanistic differences^5^. Likewise, in the setting of cancer, the reasons to use glucocorticoids in the anticancer regimen and the accompanying mechanisms may deviate: for different cancer types, activating the glucocorticoid receptor can offer benefit in very different ways. To give an example, in the struggle against hematological cancers, glucocorticoids are not part of a comfort-enhancing anticancer strategy, but are an actual cornerstone and prime driver to directly kill off the nonsolid cancer cells, via the programmed cell death mechanism (since long termed apoptosis)^6^. In solid malignant tumors, the role of the glucocorticoid receptor may range from beneficial to detrimental, and varies with the cancer type, the levels of the receptor, and the degree of crosstalk with other nuclear receptor family members that may drive a particular cancer (e.g., estrogen receptor in breast cancer, androgen receptor in prostate cancer)^7^.

Even though glucocorticoids’ ability to relieve serious symptoms of chemotherapy should be considered a crucial aspect of the anticancer treatment from a humane perspective, a concomitant reduction in antitumor activity due to the presence of glucocorticoids is unacceptable in the long run. Efforts to elucidate the mechanism behind cisplatin resistance in the presence of glucocorticoids are important to improve anticancer therapies.

In an elegant and insightful study in this issue, Pan and colleagues^8^ investigated how glucocorticoid receptor activation contributes to platinum resistance in human cancers and used a series of preclinical studies and analyses of platinum-treated patient tumor specimens. Their data reveal a hormone-independent mechanism of glucocorticoid receptor activation by direct binding to cisplatin. Cisplatin-bound glucocorticoid receptor causes the receptor to accumulate in the nucleus. This unexpected event next enhances gene expression and subsequent activation of a kinase called MAST1 (Fig. 1), a known platinum resistance factor. Indeed, active MAST1 circumvents cisplatin-mediated inhibition of the MAP kinase (MAPK) pathway and as such reactivates cancer cell growth^9^. The MAST1 inhibitor, Lestaurtinib, was shown to block this cancer-promoting pathway^9^. By tying those mechanisms together, a solid explanation is provided for the occurrence of cisplatin resistance observed with glucocorticoid treatment. Pan et al.^8^ further deepen the mechanistic insight by pinpointing the exact amino acid that is involved in the cisplatin-induced glucocorticoid receptor activation step (Fig. 1).

**Fig. 1:**
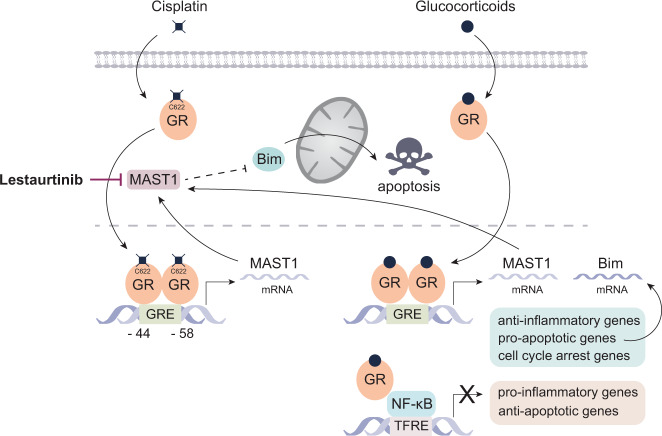
Model depicting how platinum drug therapy resistance in solid cancers involves direct glucocorticoid receptor activation and subsequent MAST1 activation. The intracellular pathway established specifically upon cisplatin-resistance and in presence of glucocorticoids is presented. Glucocorticoids (black circle) activate the glucocorticoid receptor (GR), an intracellular receptor that, when bound to ligand, can translocate to the nucleus (pathway on the right-hand side). There, the glucocorticoid receptor regulates its target genes via different mechanisms, directly or indirectly, and in a cell-type specific manner. Depicted are likely mechanisms, but alternative mechanisms are not excluded. Generally, pro-apoptotic gene expression (e.g. Bim), cell cycle arrest and anti-inflammatory gene expression are promoted, while pro-inflammatory (NF-kB-dependent) gene expression and anti-apoptotic gene expression are dampened. Besides glucocorticoid receptor monomer and dimer-driven mechanisms, also other mechanisms have been described to account for the indicated gene profiles (not depicted here). Cisplatin-resistance involves binding of cisplatin to the glucocorticoid receptor at Cystein 622, followed by its nuclear accumulation and its ability to enhance gene and protein expression of MAST1 (pathway on the left-hand side). On its turn, MAST1 can block the pro-apoptotic Bim, hereby establishing cisplatin-resistance. Addition of the MAST1 inhibitor Lestaurtinib relieves the brake on Bim and may restore sensitivity to platinum-based drugs. Lestaurtinib does not seem to affect the capacity of the glucocorticoid receptor to regulate other typical target genes, including those involved in the relief of side effects (not depicted here). Abbreviations: GR, glucocorticoid receptor, GRE, glucocorticoid response element, MAST1, microtubule-associated Ser/Thr kinase, TFRE, transcription factor response element.

What remains to be clarified is what the molecular basis may be for GR to be (preferentially) recruited to the MAST1 promoter, following a cisplatin triggering. One hypothesis is that cisplatin might induce a posttranslational modification and/or a conformational change in the glucocorticoid receptor it helps driving into the nucleus in a differential manner compared to glucocorticoids, hereby generating a glucocorticoid receptor that potentially engages in different protein–protein interactions (f.i. its chaperones or transcriptional cofactors). This cisplatin-induced alter ego of the glucocorticoid receptor could next be recruited in a more optimal way to the glucocorticoid response element identified within the MAST1 promoter. The glucocorticoid receptor is after all a multidomain transcription factor with many possibilities for posttranslational modifications. Some of those have already been linked to a changed shuttling behavior of the receptor between the cytoplasm and nucleus consequent to an active MAPK signaling pathway^10^. The glucocorticoid receptor is further known to be subject to both orthosteric and allosteric regulations^11^. Arguing for a remarkable specificity in the mechanism is that, in the Kang study, mutation of Cys622, an amino acid in the ligand-binding domain of the glucocorticoid receptor, did halt cisplatin-mediated nuclear accumulation and resistance, but left conventional glucocorticoid-mediated nuclear accumulation and target gene regulation unhampered. What kind of equilibrium is reached between cisplatin and glucocorticoids in binding the glucocorticoid receptor, and what level of multimerization this may entail, if any, remains to be studied. A long-standing conundrum in glucocorticoid receptor research is what gene-regulatory activities are mediated by monomers, dimers, or tetramers^12^.

With a compelling hypothesis and sufficient mechanistic evidence in hand to link cisplatin to the glucocorticoid receptor, the team went on to study whether the MAST1 inhibitor, Lestaurtinib, could resensitize tumor cells to cisplatin while preserving alternative supported pathways and thus potentially beneficial effects of the glucocorticoid receptor. Using two different patient-derived xenograft (PDX) cancer models in rodents, head and neck squamous cell carcinoma, and ovarian PDX, in vivo tumor growth was halted. Advantageously, Lestaurtinib add-on treatment did not affect the glucocorticoid-driven and glucocorticoid receptor-mediated overall anti-inflammatory profile.

In an independent effort to solve the glucocorticoid chemoresistance phenomenon, two other recent studies delivered compelling data for the involvement of another signaling cascade, namely the hippo pathway^13,14^. A recent other study identified in a loss-of-function screening the serum- and glucocorticoid-inducible kinase as an alternative (targetable) culprit mediating platinum drug resistance^15^. Even though a link was not investigated, the latter study might be an indirect mechanism potentially enhanced by glucocorticoid co-treatment. Collectively, these findings echo the versatility of the glucocorticoid receptor and its ability to use a diverse set of mechanisms towards different outcomes, tipping the balance either in favor or disfavor of an effective anticancer action. Important aspects that remain to be investigated in the future, upon adding an extra component to the drug cocktail, are optimal dosing, drug–drug effects, best ratios, and ideal timing. This sounds easier said than done, considering that glucocorticoids can already by themselves elicit dose-specific responses due to differential expression of glucocorticoid receptor variants, of their cofactors across different cell types^16^.

As with any chemotherapy regimen, vigilance will remain key because therapy resistance may not be present at onset, but may emerge gradually. The better all possible killing-evasion mechanisms are characterized, the more ammunition becomes available to keep glucocorticoids aboard for their much-appreciated adverse events-relieving capacity.
